# Thermoelectric quantum oscillations in ZrSiS

**DOI:** 10.1038/ncomms15219

**Published:** 2017-05-23

**Authors:** Marcin Matusiak, J. R. Cooper, Dariusz Kaczorowski

**Affiliations:** 1 Institute of Low Temperature and Structure Research, Polish Academy of Sciences, PO Box 1490, 50–950 Wrocław, Poland; 2 Cavendish Laboratory, Department of Physics, University of Cambridge, Cambridge CB3 OHE, UK

## Abstract

Topological semimetals are systems in which conduction and valence bands cross each other and the crossings are protected by topological constraints. These materials provide intriguing tests for fundamental theories, while their unique physical properties promise a wide range of possible applications in low-power spintronics, optoelectronics, quantum computing and green energy harvesting. Here we report our study of the thermoelectric power of single-crystalline ZrSiS that is believed to be a topological nodal-line semimetal. We show that the thermoelectric power is an extremely sensitive probe of multiple quantum oscillations that are visible in ZrSiS at temperatures as high as 100 K. Two of these oscillations are shown to arise from three- and two-dimensional electronic bands, each with linear dispersion and the additional Berry phase predicted theoretically for materials with non-trivial topology. Our work not only provides further information on ZrSiS but also suggests a different route for studying other topological semimetals.

## Abstract (web summary)

Studies of quantum oscillations provide a valuable probe into the electronic structure of topological semimetals. Matusiak *et al*. demonstrate that quantum features in ZrSiS can be probed with greater sensitivity using diffusive thermopower than magnetization and electrical resistivity approaches.

Among three-dimensional (3D) systems with non-trivial topological states, topological nodal-line (TNL) semimetals, in which Dirac band crossings occur along a line or form a loop in momentum space, attract much attention due to their particularly interesting physics^1^. The representatives of this type of material, examined by angle-resolved photoemission spectroscopy (ARPES), are PtSn_4_ (ref. 2), PbTaSe_2_ (ref. 3), ZrSiS^4^,^5^,^6^, ZrSiSe and ZrSiTe^7^, and HfSiS^8^. As an outcome of detailed ARPES studies on ZrSiS, this compound has been shown to harbour not only a 3D TNL phase with a diamond-shaped Fermi surface near the Brillouin zone centre but also multiple Dirac cones dispersing linearly over an extended energy range up to 2 eV (refs 4, 5, 6). Furthermore, the material hosts linearly dispersive surface states protected by the non-symmorphic crystal symmetry^4^,^5^, and is therefore an excellent candidate for the realization of a 2D topological insulator^9^,^10^.

High-quality single crystals of ZrSiS are stable in air, and can be grown from relatively abundant, non-toxic elements by chemical vapour transport^4^. The unique electronic structure of the compound makes it appropriate for comprehensive studies of electrical transport properties, anticipated to reflect the non-trivial character of the bulk states. In accordance with expectations, the magnetoresistance (MR) in ZrSiS has been found to be extremely large, non-saturating in strong magnetic fields and highly anisotropic^6^,^11^,^12^,^13^. However, these features can be interpreted in terms of the semi-metallic nature of this compound with nearly perfect electron–hole compensation and the presence of open orbits at the Fermi surface, that is, without invoking Dirac states^11^. On the other hand, the MR measurements have also revealed the occurrence of Shubnikov–de Haas (SdH) oscillations, discernible in fields as low as 2 T and at temperatures as high as 20 K (refs 6, 11, 12, 13). This finding implies very high mobility of the charge carriers. Fast Fourier transformation (FFT) of the experimental data yielded two characteristic frequencies of about 14 and 238 T, attributed to a small electron and a larger hole Fermi pocket, respectively, both with the effective mass of the charge carriers close to 0.1 *m*_0_ (*m*_0_ stands for the free electron mass)^12^. The nature of the TNL phase in ZrSiS has recently been probed also via de Haas–van Alphen (dHvA) oscillations^14^. These studies confirmed the presence of 3D Dirac states, with a dHvA frequency of about 240 T, high mobility and small effective mass of the hole-type carriers. In addition, a small electron-type Fermi pocket of 2D character has been found that probably corresponds to the Dirac surface states seen by ARPES.

Here we demonstrate that measurement of the thermoelectric power is a powerful experimental tool in capturing Dirac states in TNL semimetals. We are able to detect five independent quantum oscillations and investigate phase shifts for three of them. The results obtained for ZrSiS provide further information on the multiple Dirac bands evidenced in the ARPES experiments on this compound and trace a different path for studying related Dirac materials.

## Results

### Zero magnetic field thermoelectric properties

**Figure 1:**
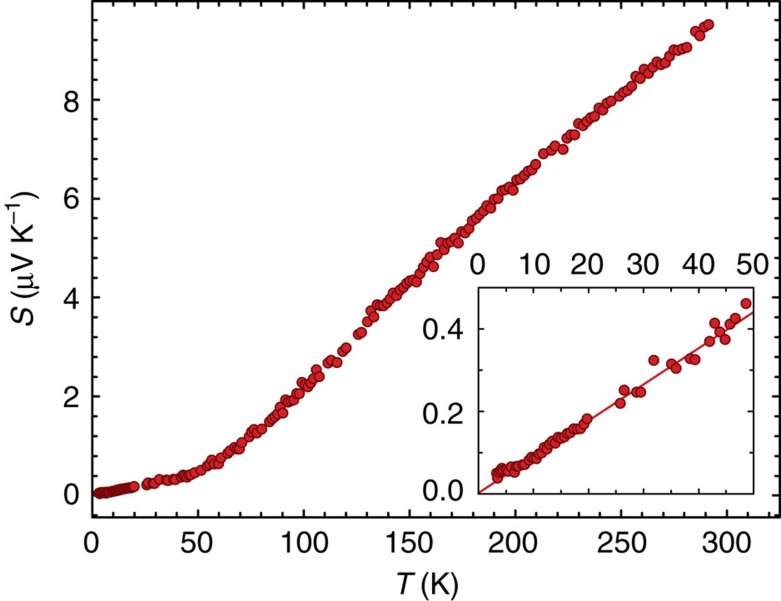
The temperature dependence of the *a* axis thermoelectric power for ZrSiS in zero magnetic field. Inset shows the low temperature part of the same data and a linear fit through the origin.

Despite ZrSiS being dubbed a semi-metal, its thermoelectric power (*S*), as well as the electrical resistivity (*ρ*)^12^, measured in the absence of magnetic field have a form similar to those for metals. Figure 1 shows that *S* has a rather moderate value at the room temperature (around 10 μV K^−1^ at 300 K). It stays positive in the entire temperature (*T*) range and varies almost linearly with *T*. The change in slope around *T*≈50 K is unlikely to be caused by a phonon-drag contribution, despite the high quality of the crystals studied, because the low-temperature part of *S*(*T*) shown in the inset in Fig. 1 remains linear, in contrast to the *T*^3^ dependence expected from phonon-drag^15^. Perhaps the natural variation in isotope composition for the constituent elements^16^ enhances phonon–phonon scattering and suppresses phonon drag. Also, large angle phonon–electron scattering, which is needed to transfer the phonon momentum to the electrons efficiently, will be small at low *T* when typical phonon wave vectors are large compared to the Fermi wave vectors (*k*_F_). As shown in more detail in the Supplementary Information, the low values of the quantum oscillation frequencies (*F*) show that several carrier pockets are sufficiently small at 20 K. We note in passing that the analogous effect for electron–phonon scattering provides a natural explanation for the *T*^*3*^ dependence of the electrical resistivity observed at low *T* (ref. 12). Namely, the extra factor of *T*^*2*^ arising from large angle electron–phonon scattering that leads to a *T*^*5*^ law in the usual Debye theory, will be absent for small pockets of charge carriers.

### Thermoelectric power in a magnetic field

**Figure 2:**
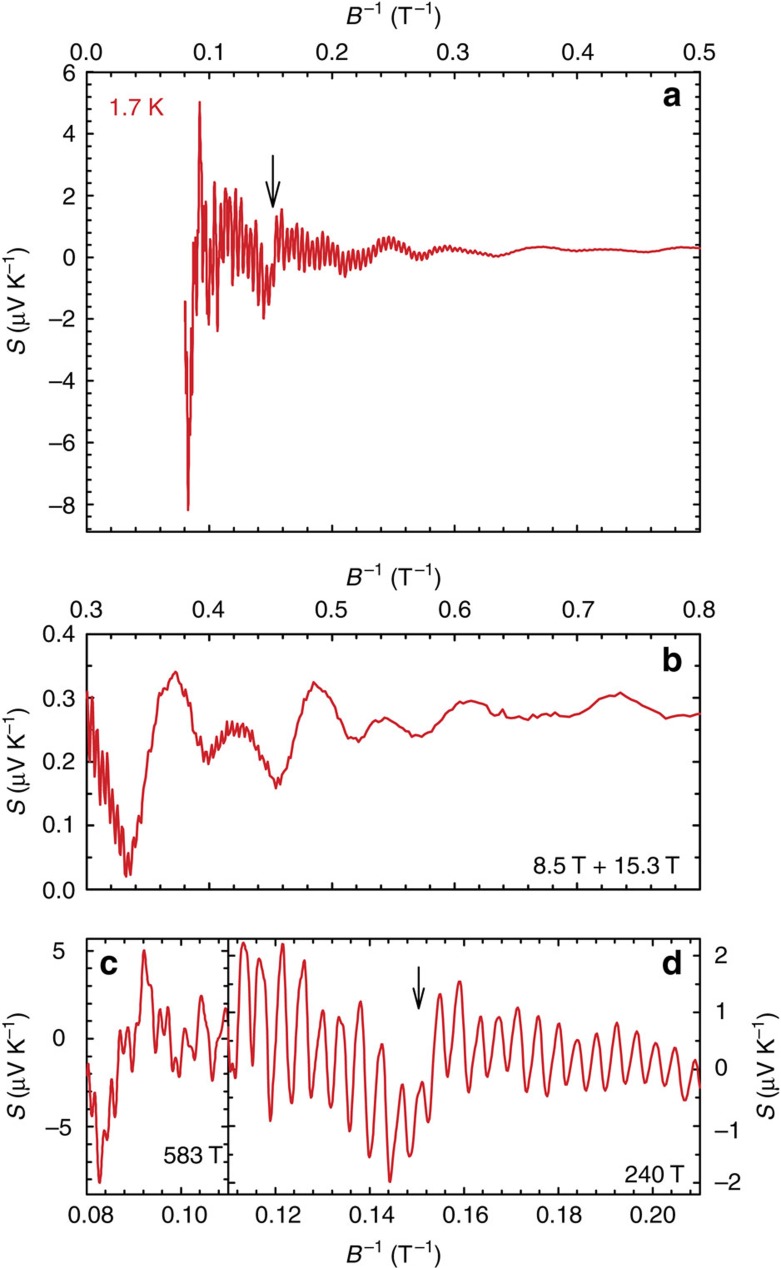
The magnetic field dependence of the thermoelectric power in ZrSiS at *T*=1.7 K. Panel (**a**) presents a broad view, whereas panels (**b**–**d**) show the same data on different scales chosen to expose distinct oscillation frequencies—the corresponding values of *F* (in Tesla) are indicated. The arrows mark a jump at *B*^−*1*^*=*0.15 *T*^−*1*^, as discussed in the text.

**Figure 3:**
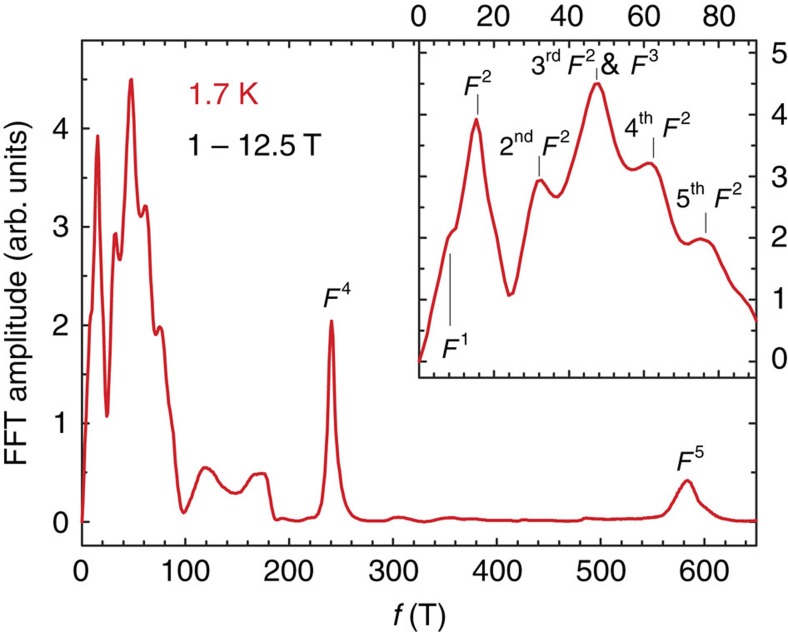
The FFT spectrum calculated for the thermoelectric power data at *T*=1.7 K. Inset shows the low-frequency part, where peaks are attributed to *F*^1^, *F*^2^ and *F*^3^ frequencies as well as higher harmonics of *F*^2^.

**Figure 4:**
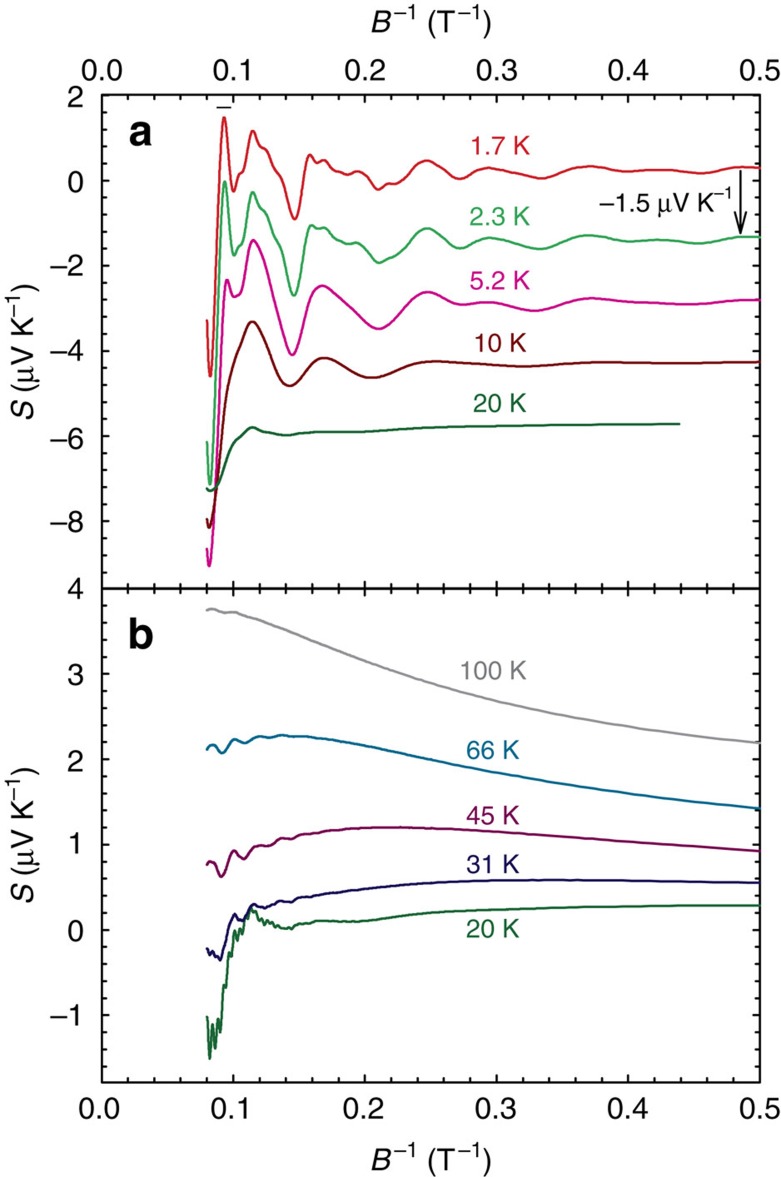
The magnetic field dependences of the thermoelectric power in ZrSiS at selected temperatures. Upper panel (**a**) shows low temperature (*T*≤20 K) data (shifted vertically by 1.5 μV K^−1^ for clarity), where high frequency oscillations were filtered out. ‘−’ and ‘+’ symbols mark Zeeman splitting. Bottom panel (**b**) shows the raw high-temperature data (*T*≥20 K).

**Figure 5:**
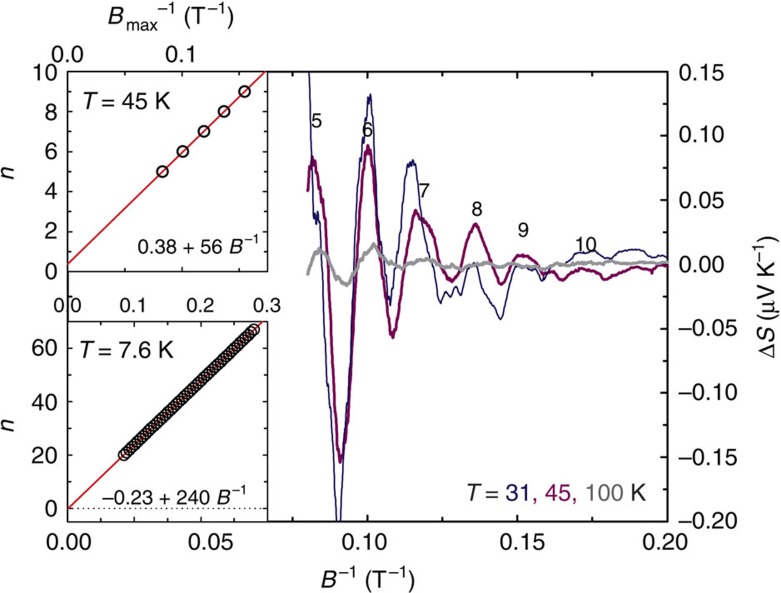
The magnetic field dependence of the oscillatory part of the thermoelectric power in ZrSiS at high temperature. Insets show the Landau-level index plot for *F*^3^ (upper one, 57 T) and *F*^4^ (bottom one, 240 T) together with parameters of the linear fits.

While the *S*(*T*) dependence of ZrSiS appears to be simple for a material with a multiband electronic structure^4^,^5^ and low-charge carrier densities^12^, application of a magnetic field along the crystallographic *c* axis reveals unusual properties. One indication of these is the enormous MR^11^,^12^,^13^, another is the wide temperature and magnetic field range where quantum oscillations are observed. Figure 2 shows the field dependence of the thermoelectric power of ZrSiS measured at *T*=1.7 K. As one can easily judge, the signal is completely dominated by the oscillatory part that reaches a value of ∼8 μV K^−1^ at this temperature. Observations of dHvA and SdH oscillations in ZrSiS have been reported recently^13^,^14^, but we find that the thermoelectric response is much more sensitive to these oscillations. This has been found valid for a number of other compounds^17^,^18^. We are able to detect five different oscillations with frequencies: *F*^1^=8.5, *F*^2^=15.3, *F*^3^=57, *F*^4^=240 and *F*^5^=583 T. They can be seen in Fig. 3, which shows the FFT spectrum calculated for data collected at *T*=1.7 K in the 1–12.5 T magnetic field range (here *F*^3^ is likely merged with the third harmonic of *F*^2^). Because of the exceptional sensitivity of thermoelectric effects to quantization of the Fermi surface mentioned above, we can observe the *F*^3^ and *F*^5^ oscillations that have not been reported before. The *F*^1^, *F*^2^, *F*^4^ and *F*^5^ oscillations are immediately visible in the different field regions of the *S*(*B*^−1^) plots shown in the panels (b), (c) and (d) of Fig. 2. However, detailed analysis is not straightforward because of the presence of multiple frequencies and their higher harmonics. Figure 4 shows *S*(*B*^−1^) plots for selected temperatures, where data in the upper panel has been averaged to remove frequencies higher than ∼200 T while the bottom panel show raw (non-filtered) high-temperature data. Noticeably, in the high-field limit, oscillations with frequency *F*^3^=57 T are still present at *T* as high as 100 K (this is more evident in Fig. 5). As the temperature is lowered, the oscillations become more pronounced but at the same time less and less regular. It has been suggested that a saw-tooth shape (and therefore high-harmonic content) is a typical feature of quantum oscillations in *S* (ref. 17).

### Irregularities in *S*(*B*)

**Figure 6:**
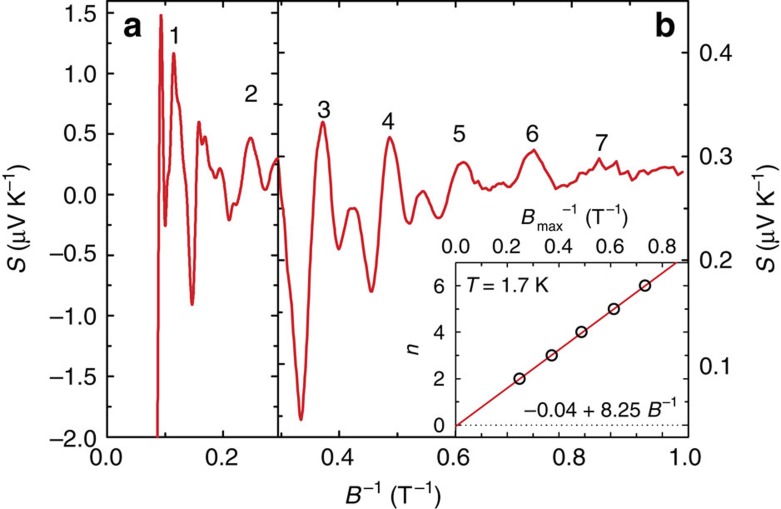
The magnetic field dependence of the filtered (low frequency) thermoelectric power data in ZrSiS at 1.7 K shown on different vertical scales. The scale for panel (**a**) is −2 to 1.6μV/K and for panel (**b**) is 0.04 to 0.45μV/K. Inset shows the Landau level index plot for *F*^1^ (8.5 T) together with parameters of the linear fit.

It is difficult to judge definitely whether the apparent irregularities in *S*(*B*^−1^) dependences at low *T* are a consequence of a growing contribution from higher harmonics or of a different origin. For instance, the sudden jump in *S* at *B*^−1^≈0.15 T^−1^ for *T*=1.7 K marked with an arrow in Fig. 1, and which can also be seen in the filtered data shown in Figs 4 and 6, can be an extreme effect of the higher harmonics. However, *B*^−1^≈0.15 T^−1^ also seems to be the field where the highest frequency oscillation (*F*^5^) arises and the step in *S*(*B*^−1^) could be a sign of a magnetic breakdown. Intriguingly, *B*^−1^≈0.15 T^−1^ is also the field, where the high-temperature *F*^3^ oscillation (that dominates the high-temperature oscillatory signal) emerges. However, magnetic breakdown is expected to give rise to sum or difference frequencies for type I (ref. 19) or type II (ref. 20) Weyl semimetals, respectively. There is no clear evidence for sum or difference frequencies involving either *F*^3^ or *F*^5^, so therefore, the step-like change in *S*(*B*^−1^) probably results from higher harmonics of *F*^2^. The clear splitting of the peak at *B*^−1^≈0.1 T^−1^ could be associated with the Zeeman effect that was suggested by dHvA studies to be particularly prominent in ZrSiS^14^. The Landé *g* factor calculated for *F*^1^ from the energy difference between the spin-split Fermi surfaces, Δ_*S*_=gm*/4*m*_0_=½(*F*/*B*_+_−*F*/*B*_−_)^21^ (m* is the cyclotron mass of charge carriers that is given by the energy dependence of the extremal Fermi surface areas that are proportional to the oscillation frequencies *F*, while the signs + and − represent the split peaks), is *g*≈6. This is smaller than value reported in ref. 14, partly because the estimated effective masses are different.

A feature that is unlikely to be related to the emergence of higher harmonics is the sudden drop of *S* in the high-field limit (*B*^−1^<0.093 T^−1^). This anomaly broadens at higher temperatures, but can still be observed even at *T*=66 K, where higher harmonics of *F*^3^ are long gone. Here it is worth noting that for the *B*>10 T (*B*^−1^<0.1 T^−1^), we reach the quantum limit for the *F*^1^ band, that is, the first Landau level crosses the Fermi energy at *B*^−1^≈0.11 T^−1^ as implied by the Landau-level plot in the inset to Fig. 6. Similar behaviour of the thermoelectric power while crossing from the *n*=1 to the *n*=0 Landau level was reported as evidence for massive bulk Dirac fermions in Pb_1−*x*_Sn_*x*_Se (ref. 22).

## Discussion

**Figure 7:**
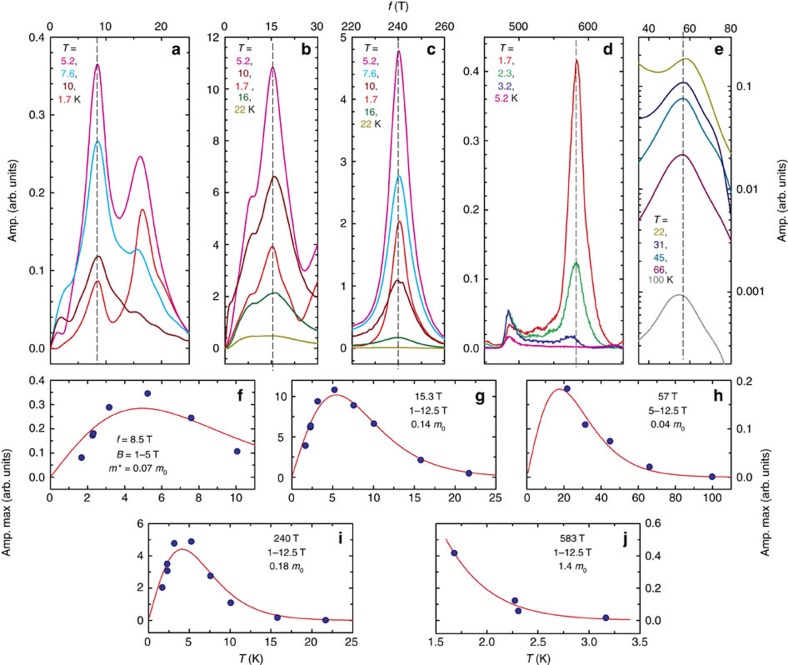
The temperature dependences of the peak heights obtained from the FFT. The top panels (**a**–**e**) show FFT spectra calculated for various temperatures, the bottom panels (**f**–**j**) show the temperature dependence of the peak heights determined from the corresponding FFT plots. Solid red lines in the bottom panels are fits to equation (1).

As mentioned above, the *F*^1^, *F*^2^ and *F*^4^ oscillations were already seen in previous studies, but intriguingly, while *F*^4^ was observed in both dHvA (240 T (ref. 14)) and SdH (238 T (ref. 12), 246 T (ref. 6), 243 T (ref. 13)) measurements, *F*^1^ was only detected in dHvA (8.4 T (ref. 14)), and *F*^2^ only in SdH (14.1 T (ref. 12), 18.9 T (ref. 6)). Our study shows that while both *F*^1^ and *F*^2^ are present, *F*^1^ is relatively broad and generally overlaps with *F*^2^, which is almost a factor of 2 higher. On the other hand, *F*^1^ and *F*^2^ differ in their temperature and field dependences—for example, when *B*^−1^>0.5 T^−1^ at *T*=1.7 K only *F*^1^ persists, as shown in the main part of Fig. 6. Therefore, we determine the temperature dependences of the FFT peak amplitudes in three different field regions, 1–12.5, 1–5 and 5–12.5 T, depending on the range where a given frequency is more visible. Figure 7 shows the results of this procedure, where one can see that the evolution of the FFT peak amplitudes clearly differs from the Lifshitz–Kosevich formula that predicts maximal amplitude at zero temperature^23^. This formula cannot be directly applied to oscillations in the thermoelectric power, which, being related to the entropy carried by quasiparticles and their heat capacity, is expected to vanish in the zero temperature limit. Therefore, we use the formula for quantum oscillations in *S* applied recently by Morales *et al*.^24^ to analyse data on UGe_2_:


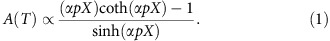


As the temperature is lowered, *A(T)* rises to a maximum at 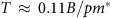 and approaches zero for *T*→0 K. Here 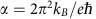 (*k*_B_ is the Boltzmann constant, *e* is the elementary charge, *ħ* is the reduced Planck constant), *p* is the harmonic number and 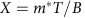. Fits of the data to equation (1) shown in the lower panels of Fig. 7 give the cyclotron masses summarized in Table 1.

The only band with an effective mass comparable to *m*_*0*_ is *F*^5^, whereas for the others, m* is one—two orders of magnitude smaller. The values obtained here for *F*^1^, *F*^2^ and *F*^4^ are in good agreement with previously reported results except for estimates given by Hu *et al*.^14^ that are two–three times smaller. The lightest charge carriers originate from the *F*^1^ and *F*^3^ bands (m*=0.07*m*_0_ and 0.04*m*_0_, respectively) so it is especially interesting to see whether the phases of these oscillations exhibit non-trivial properties corresponding to Dirac fermions. To do so, we made Landau-level fan diagrams, shown as insets in Fig. 5 (*F*^3^) and Fig. 6 (*F*^1^). Here the integers (*n*) are determined by the values of *F* found from FFTs together with the positions of the peaks from plots of *S* versus *B*^−1^. The low-frequency *F*^1^ and *F*^3^ oscillations give peaks at low integers approaching to the origin of the plots. We also prepared the Landau-level fan diagram for *F*^4^ (shown in the lower inset in Fig. 5) for comparison with previous works. Having found the values of *n*, all data, apart from *F*^2^ and *F*^5^ that were excluded due to the small number of maxima available for indexing, were then fitted to straight lines with two free parameters, where, as shown in the figures, the resulting slopes match well with the quantum oscillation frequencies determined from FFT. We obtain the extrapolated phase shift (*ϕ*_S_) of *F*^4^, *n*(*B*^−*1*^=0)=−0.23±0.02. As explained below, this agrees perfectly with values obtained by Wang *et al*.^6^ and Ali *et al*.^13^ from SdH measurements (see Supplementary Table 1), even though the measured values differ by 0.25. This is because quantum oscillations in the diffusion thermoelectric power are shifted by *π*/2 (or *δn*=1/4) in relation to SdH^25^,^26^,^27^,^28^, which means that the equivalent phase shift expected for SdH is given by *ϕ=ϕ*_S_±1/4. This *π*/2 phase shift arises because the diffusion thermoelectric power depends on how the electron or hole density of states changes with energy. According to the Mott formula^29^, *S* is related to the logarithmic derivative of the electrical conductivity (*σ*) with respect to energy (*ε*) at the chemical potential (*μ*) by:


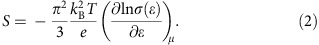


Taking into consideration the sign of the charge carriers, we conclude that the shift is +*π*/2 for electrons and −*π*/2 for holes. Indeed, in graphene a *π* change in phase was observed between the thermoelectric power and the resistivity when the sign of the gate voltage was reversed^30^. This confirms that the sign of the phase shift for *S* depends on the sign of the charge carriers. We do not observe any variation in the phase of the oscillations with the magnetic field, showing that *S* remains in the so-called Mott regime^28^. Making the usual assumption that SdH oscillations arise because changes in *σ* are proportional to oscillations in the density of states, then the total phase shift *ϕ* in the Lifshitz–Kosevich formula^23^ describing the SdH effect: cos[2*π*(*F*/*B*+*ϕ*)] is believed to be **±**1/2 and **±**5/8 for 2D and 3D parabolic bands, respectively (+ for holes and − for electrons)^31^. In contrast, the values *ϕ*=0 and **±**1/8 are expected for 2D and 3D Dirac cones (although it could also be **±**5/8 for the latter^31^) because of the additional Berry phase *π* that is accumulated by Dirac fermions along cyclotron orbits^32^,^33^). The phase shifts measured in the thermoelectric power and summarized in Table 1, are: *ϕ*_*S*_*=*−0.23±0.02 (−1/4), 0.38±0.03 (3/8), and 0.04 **±**0.04 (0), (in units of *n*, that is, 2*π*) for *F*^4^, *F*^3^ and *F*^1^, respectively (see the Supplementary Information for more details).

Applying the above formula, *ϕ*=*ϕ*_S_ **±**1/4, where the + sign refers to electrons and the − sign to holes, to our thermoelectric data for *F*^4^ gives *ϕ*=**−**1/4+1/4=0, which is expected for a 2D electron-like Dirac cone^31^. SdH work in refs 6, 13 gives the same value of *ϕ* but no information on the sign of the charge carriers. 14 gives a different value of *ϕ* but here the value of m* is also different, as shown in Table 1. Similarly for *F*^3^, for which there is no SdH data: *ϕ*=3/8−1/4=1/8. We note that *ϕ*_S_=3/8 can only arise from a 3D hole-like Dirac cone. It cannot arise from a normal 3D parabolic band because *ϕ*=5/8 would require inconsistent signs of the carriers. Namely, in the relations: *ϕ*=5/8=3/8+1/4, the 5/8 is hole like while the +1/4 is electron like. However, for *F*^1^, the above formula gives *ϕ*=0±1/4=**±**1/4, which is rather unexpected.

Therefore, we conclude that *F*^3^, with the phase shift *ϕ* being exactly 1/8, very small effective mass, and a high mobility that allows oscillations to be visible even at *T*=100 K, is a textbook example of 3D Dirac fermion behaviour. We also indicate that *F*^4^ is a 2D Dirac cone, which is consistent with the electronic structure calculations^4^,^5^,^6^,^11^, ARPES results^4^,^5^ and SdH data^6^,^12^,^13^. On the other hand, the phase shift determined for *F*^1^ does not match values suggested by present theoretical models and remains puzzling. Other interesting parameters deduced from our work, namely the values of the spanning vector 2*k*_F_, compared with a typical phonon wave vector, the carrier concentration in a 2D model, for comparison with experimental and theoretical work on the 2D electron gas^34^,^35^, and the Fermi energy relative to the bottom of each band, are summarized in the Supplementary Information.

In summary we show that the thermoelectric power is a powerful tool for studying the complex quantum properties of the ZrSiS nodal-line semimetal. We distinguish five different oscillations in *S*(*B*^−1^) with frequencies from *F*^1^=8.5 T to *F*^5^=583 T, whereas measurements of the dHvA and SdH effects were only able to detect two of them simultaneously. By applying the Lifshitz–Kosevich formula modified for the thermoelectric power for all five extremal orbits, we determined their effective masses, which for *F*^1^, *F*^2^, *F*^3^ and *F*^4^ turned out to be one or two orders of magnitude smaller than the free electron mass. Determining the phase shifts allowed us to conclude that *F*^3^ and *F*^4^ oscillations originate from hole-like 3D and electron-like 2D Dirac cones, respectively. On the other hand, we do not find a good explanation for the zero shift in *ϕ*_*S*_ measured for *F*^1^. This low-frequency oscillation appears to reach the quantum limit when the magnetic field is higher than 10 T.

## Methods

### Sample synthesis

The single crystals of ZrSiS used in the present research were selected from the same batch as those studied before by ARPES^5^. They were grown by a chemical vapour transport method described elsewhere^11^. More details regarding sample quality are presented in the Supplementary Information.

### Thermoelectric power measurements

The thermoelectric power (*S*) was measured along the *a* axis of a single crystal of dimensions 1.7 × 1.7 × 0.12 mm^3^ with magnetic field (*B*) parallel to the *c* axis. A sample was clamped between two phosphor bronze blocks, to which two Cernox thermometers and resistive heaters were attached. The experimental rig was calibrated against a high-temperature superconductor (at low *T*) and a pure Pb sample (at high *T*) to allow subtraction of the contribution from the leads, which turned out to be small (∼0.1 μV K^−1^). The thermal gradient was about 10% of *T* or lower, and the thermoelectric voltage varied linearly with Δ*T* (see Supplementary Fig. 2). The voltage signal was measured at a given temperature during a field sweep from −12.5 to +12.5 T and vice versa, to exclude a small component of the thermoelectric effect that was antisymmetric in *B*.

### Data availability

The data that support the findings of this study are available from the corresponding author on request.

## Additional information

**How to cite this article:** Matusiak, M. *et al*. Thermoelectric quantum oscillations in ZrSiS. *Nat. Commun.* **8,** 15219 doi: 10.1038/ncomms15219 (2017).

**Publisher’s note**: Springer Nature remains neutral with regard to jurisdictional claims in published maps and institutional affiliations.

## Supplementary Material

Supplementary Information Supplementary Figures, Supplementary Table, Supplementary Notes and Supplementary References

## Figures and Tables

**Table 1:** Comparison of effective masses of charge carriers for different bands of ZrSiS reported by various groups.

**Frequency (T)**	***F***^**1**^**: 8.5**	***F***^**2**^**: 15.3**	***F***^**3**^**: 57**	***F***^**4**^**: 240**	***F***^**5**^**: 583**
Effective mass (m*/*m*_0_), *S* (present work)	0.07	0.14	0.04	0.18	1.4
Phase shift (*ϕ*_*S*_), *S* (present work)	0.04±0.04	—	0.38±0.03	−0.23±0.02	—
m*/*m*_0_, SdH^6^	—	0.12	—	0.16	—
m*/*m*_0_, SdH^12^	—	0.1	—	0.14	—
m*/*m*_0_, SdH^13^	—	0.11	—	0.16	—
m*/*m*_0_, dHvA^14^	0.025	—	—	0.052	—
