# Supplementary material for: Thermoelectric quantum oscillations in ZrSiS
Source: Nat Commun. 2017 May 23;8:15219. doi: 10.1038/ncomms15219 (PMC5529674; doi:10.1038/ncomms15219)
Supplement: Supplementary Information — Supplementary Figures, Supplementary Table, Supplementary Notes and Supplementary References [file ncomms15219-s1.pdf]

Supplementary Note 1 – sample quality

The chemical composition of ZrSiS single crystals was examined by energy dispersive x-ray (EDX) analysis using an FEI scanning electron microscope equipped with an EDAX Genesis XM4 spectrometer. The result confirmed homogeneous single-phase material with stoichiometry equal to the nominal one (see Supplementary Fig. 1). The crystallinity and the crystallographic orientation of the selected specimens were assessed by the Laue X-ray backscattering technique employing a LAUE-COS single crystal orientation system (Proto Manufacturing) equipped with a CCD camera. An example of the observed patterns is shown in the inset to Supplementary Fig. 1. Additionally, a small piece of the same crystal was examined on an Oxford Diffraction Xcalibur four-circle diffractometer. The lattice parameters derived from this experiment were in very good agreement with the literature data [1]. As a further test of the quality of the crystal chosen for our thermoelectric power study, its electrical resistivity was measured by ac four-probe technique with the current flowing within the (001) plane. The residual resistivity ρ(2 K) = 0.3 μΩcm and the residual resistivity ratio, ρ(300 K)/ρ(2 K) of about 50 show the very high quality of the selected crystal.

Supplementary Note 2 – phase shift determination

To show the robustness of determination of the phase shifts we extracted them for each studied frequency again, this time at different temperatures. Namely, for:

F^1 (8.5T) we find: ϕ_S = -0.06 ± 0.02 at T = 2.3 K compared with 0.04 ± 0.04 at 1.7 K,

F^3 (57 T): ϕ_S = 0.37 ± 0.03 at 66 K vs. 0.38 ± 0.03 at 45 K,

F^4 (240 T): ϕ_S = -0.25 ± 0.02 at 10 K vs. -0.23 ± 0.02 at 7.6 K.

To check if the latter shows a field dependence we made a linear fit for the lower half of the data set (lower values of B^{-1}), which yielded ϕ_S = -0.28 +-0.03, which shows that the effect is not significant.

Supplementary Table 1 – Characteristics of the SdH oscillations in ZrSiS

Frequency (T)	F^1: 8.5	F^2: 15.3	F^3: 57	F^4: 240	F^5: 583
Cyclotron mass m^* (m_0)	**0.07**	**0.14**	**0.04**	**0.18**	**1.4**
Phase shift	**0.04**	**-**	**0.38**	**-0.23**	**-**
2 $k_F(A^o)^{-1}$	0.032	0.044	0.084	0.17	0.267
E_F (K)	163	146	1910	1790	558
Equivalent 2D carrier concentration ($10^{11}/cm^2$)	4.1	7.4	28	117	284
Phase shifts from SdH (other works)	-	0.5 [6]	-	0.04 [6], 0.03 [7], 0.15 [8]	-
Phase shifts from dHvA (other works)	0.34 [9]	-	-	0.29 [9]	-

Table Caption

From top:

Quantum Oscillation Frequencies, F^1-F^5 determined by Fourier transforming the thermopower data over various field ranges.

Values of the effective or cyclotron mass m^* determined by fitting the temperature dependence to Eqn. 1 in text.

Values of the Fermi surface spanning vector $2k_F$ obtained by applying the formulae $A = \pi k_F^2 = 2\pi eF/\hbar$ Ref. [2] to the measured values of F. For the lowest three frequencies they are less than, or comparable with, the typical phonon momentum at 20 K, $q = 0.064\ (A^o)^{-1}$. This justifies our suggestion that for these three pockets of carriers the q^2 weighting factor associated with small angle electron-phonon scattering processes should be omitted. The initial T-dependence of the resistivity is therefore expected to be T^3 rather than the more usual T^5 behaviour for materials with a large Fermi surface. We calculated the average acoustic phonon velocity v_{av} =4.1 km/sec from the T^3 term in the low temperature heat capacity given in Ref. [3] and then calculated q using the formula $\hbar v_{av} q = k_B T$.

Corresponding values of the Fermi energy relative to the bottom of each band obtained by applying the formula $E_F = (\hbar k_F)^2/2m^*$ for a parabolic band. For a linear dispersion, $E \alpha k$, the calculated E_F values are a factor of 2 larger for the same values of m^* and k_F.

Equivalent 2D carrier concentration (n_{2D}) obtained by applying the formula, $n_{2D} = k_F^2/(2\pi)$. The two lowest values correspond to those observed in 2D systems such as heterojunctions Refs.[4,5].

Phase shifts from other work.

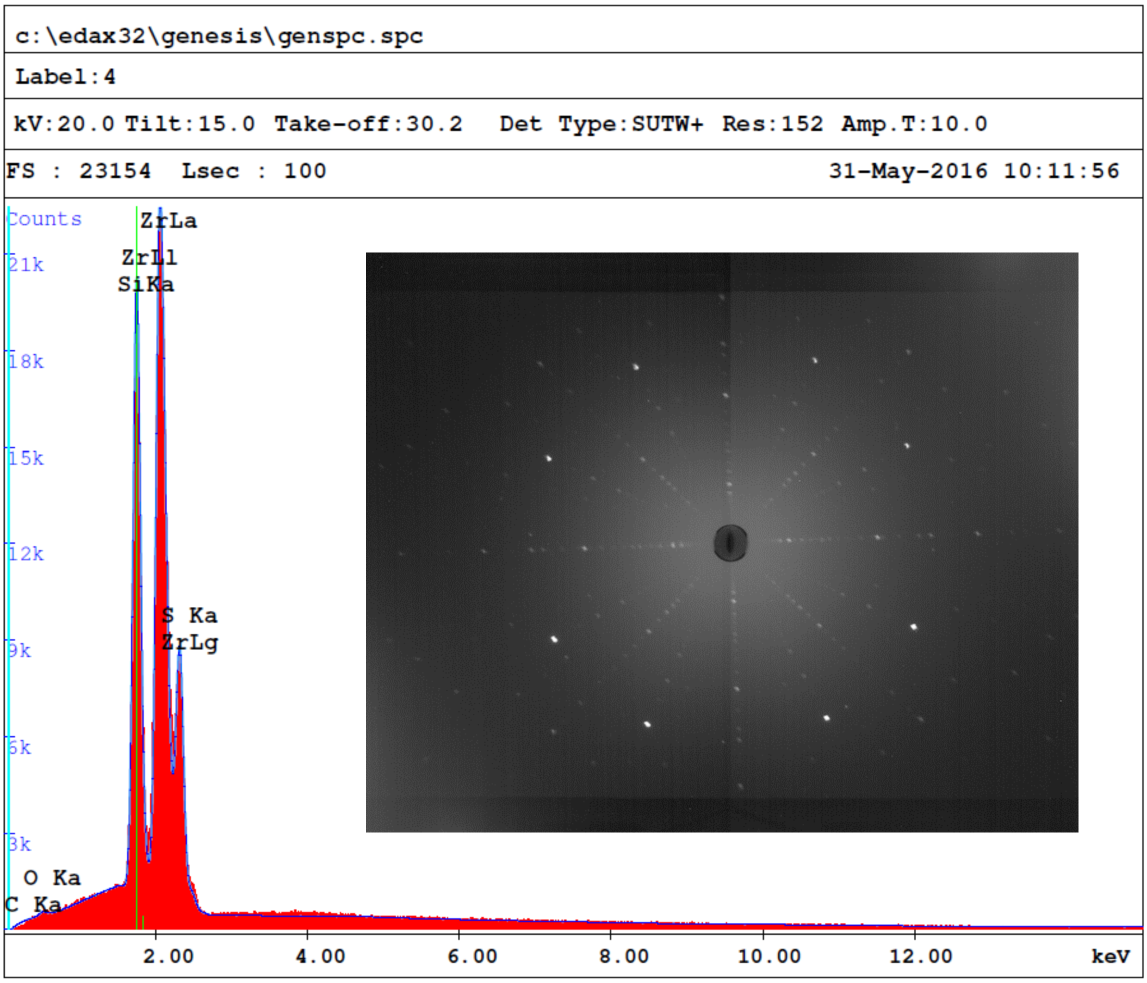

EDAX ZAF Quantification (Standardless)
Element Normalized
SEC Table : Default

Element	Wt %	At %	K-Ratio	Z	A	F
SiK	18.55	33.56	0.1639	1.1109	0.7756	1.0253
ZrL	60.91	33.92	0.4862	0.9080	0.8747	1.0050
S K	20.53	32.53	0.0964	1.1230	0.4181	1.0000
Total	100.00	100.00				

Element	Gross Inte.	Inte. Error
C K	20.85	2.19
O K	63.49	1.26
SiK	2217.71	0.21
ZrL	2644.14	0.19
S K	1137.15	0.30
ZrK	31.53	1.78

Supplementary Figure 1. The energy dispersive x-ray (EDX) spectrum obtained for the ZrSiS single crystal used in the thermoelectric power measurements and its numerical evaluation. Inset: Laue image proving a (001) orientation of the crystal surface.

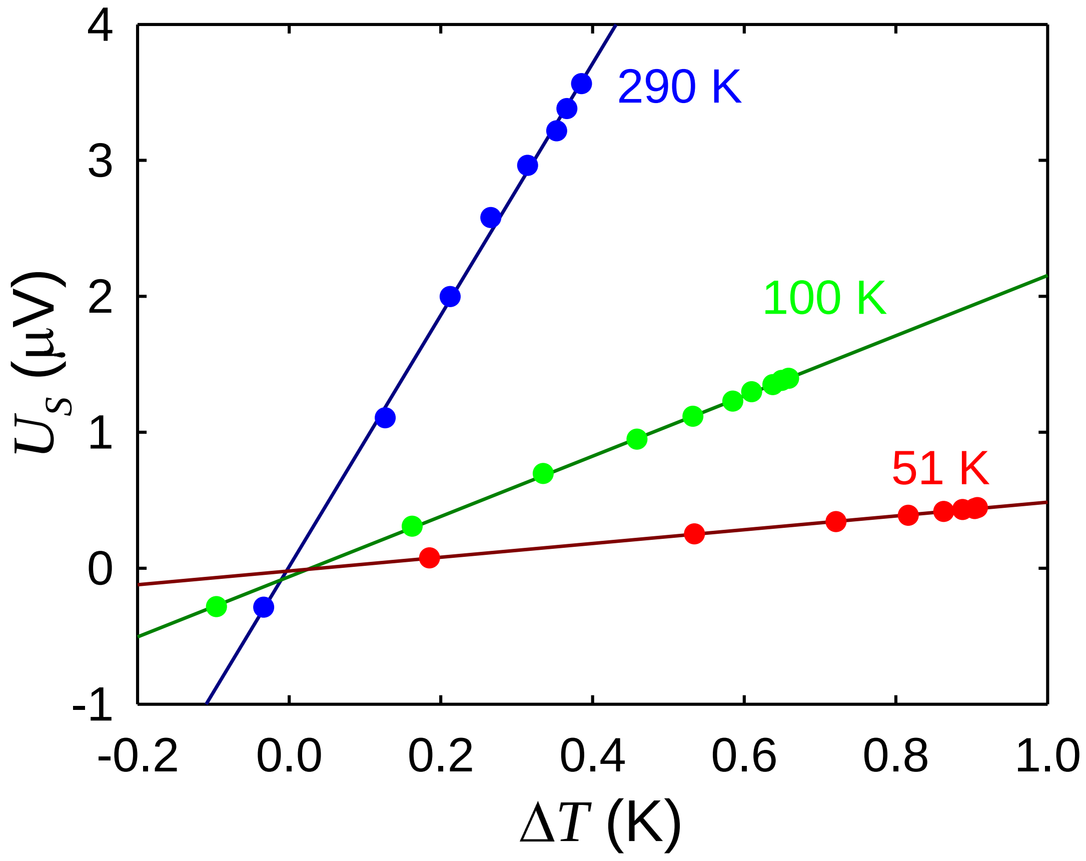

Supplementary Figure 2. Dependences of the thermoelectric signal on size of the thermal gradient across the sample at 51, 100 and 290 K.

Supplementary References

[1] Leslie M. Schoop, Mazhar N. Ali, Carola Strasser, Andreas Topp, Andrei Varykhalov, Dmitry Marchenko,Viola Duppel, Stuart S.P. Parkin, Bettina V. Lotsch and Christian R. Ast. Dirac cone protected by non-symmorphic symmetry and three-dimensional Dirac line node in ZrSiS. *Nature Commun.* **7**, 11696 (2016).

[2] D. Shoenberg. Magnetic Oscillations in Metals (Cambridge University Press, Cambridge, England, 1984).

[3] Raman Sankar, G. Peramaiyan, I. Panneer Muthuselvam, Christopher J. Butler, Klauss Dimitri, Madhab Neupane, G. Narsinga Rao, M.-T. Lin & F.C. Chou. Crystal growth of Dirac semimetal ZrSiS with high magnetoresistance and mobility. *Scientific Reports* **7**, 40603 (2017.

[4] V.M. Pudalov, M.E. Gershenson, and H. Kojima. Probing electron interactions in a two-dimensional system by quantum magneto-oscillations. *Phys. Rev. B* **90**, 075147 (2014).

[5] Tsuneya Ando, Alan B. Fowler, and Frank Stern. Electronic properties of two-dimensional systems. *Rev. Mod. Phys.* **54**, 437-672 (1982).

[6] Xuefeng Wang, Xingchen Pan, Ming Gao, Jihai Yu, Juan Jiang, Junran Zhang, Huakun Zuo, Minhao Zhang, Zhongxia Wei, Wei Niu, Zhengcai Xia, Xiangang Wan, Yulin Chen, Fengqi Song, Yongbing Xu, Baigeng Wang, Guanghou Wang and Rong Zhang. Evidence of Both Surface and Bulk Dirac Bands and Anisotropic Nonsaturating Magnetoresistance in ZrSiS, *Adv. Electron. Mater*. **2**, 1600228 (2016).

[7] Mazhar N. Ali, Leslie M. Schoop, Chirag Garg, Judith M. Lippmann, Eric Lara, Bettina Lotsch, Stuart Parkin. Butterfly magnetoresistance, quasi-2D Dirac fermi surfaces, and a topological phase transition in ZrSiS, *Science Advances* **2**, e1601742 (2016).

[8] R. Singha, A. Pariari, B. Satpati, P. Mandal. Titanic magnetoresistance and signature of non-degenerate Dirac nodes in ZrSiS. Preprint at https://arxiv.org/abs/1602.01993 (2016).

[9] Jin Hu, Zhijie Tang, Jinyu Liu, Yanglin Zhu, Jiang Wei, Zhiqiang Mao. Evidence of Dirac cones with 3D character probed by dHvA oscillations in nodal-line semimetal ZrSiS. Preprint at https://arxiv.org/abs/1604.01567 (2016).
